# Supplementary material for: Association of a genetic risk score with BMI along the life-cycle: Evidence from several US cohorts
Source: PLoS One. 2020 Sep 17;15(9):e0239067. doi: 10.1371/journal.pone.0239067 (PMC7497990; doi:10.1371/journal.pone.0239067)
Supplement: S1 Appendix — (PDF) [file pone.0239067.s001.pdf]

Association of a Genetic Risk Score with BMI along the Life-Cycle: Evidence from several US Cohorts
S1 Appendix

Anna Sanz-de-Galdeano[1,2,3*], Anastasia Terskaya[4], Angie Upegui[1],

1 FAE, University of Alicante, San Vicente del Raspeig, Alicante, Spain
2 IZA
3 CRES-UPF
4 School of Economics and Business, University of Navarra, Pamplona, Spain

* anna.sanzdegaldeano@gmail.com

Figures and tables

Fig 1. Association between BMI Polygenic Scores and BMI Along the Life-Cycle and Along the BMI Distribution. HRS Original Cohort

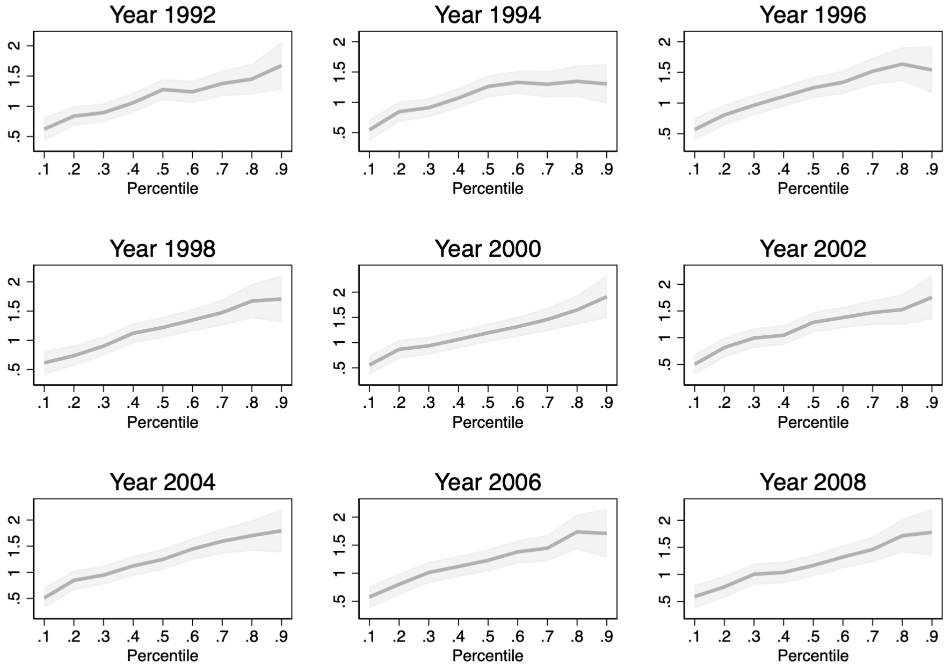

Note: Results based on our benchmark balanced panel sample of HRS Original cohort members described in Table 1 of the main text. The dependent variable is BMI. The figure shows unconditional quantile regression coefficient estimates of $BMIPGS$ (normalized to have mean 0 and standard deviation 1) in equation 2, and their associated 95% confidence intervals. All regressions include a female dummy, age, age squared, and the first 10 principal components of the full matrix of genetic data as controls. Standard errors are clustered at the household level. *** p<0.01, ** p<0.05, * p<0.1.

Fig 2. Association between BMI Polygenic Scores and BMI Along the Life-Cycle and Along the BMI Distribution. Add Health Cohort

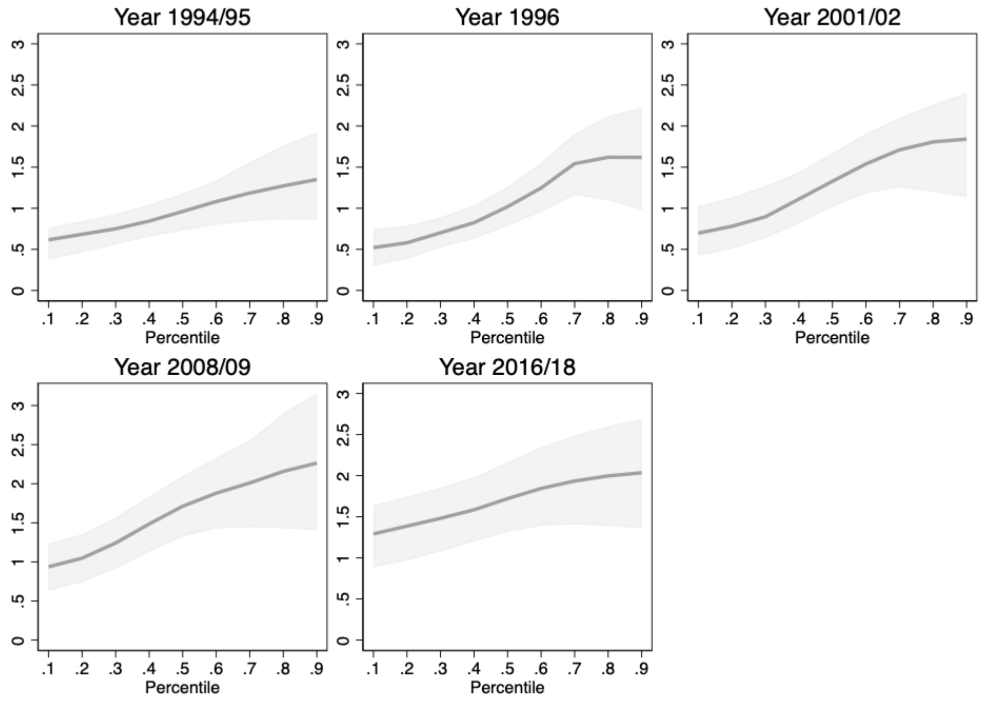

Note: Results based on our benchmark balanced panel sample of HRS Original cohort members described in Table 2 of the main text. The dependent variable is BMI. The figure shows unconditional quantile regression coefficient estimates of $BMIPGS$ (normalized to have mean 0 and standard deviation 1) in equation 2, and their associated 95% confidence intervals. All regressions include a female dummy, age, age squared, and the first 10 principal components of the full matrix of genetic data as controls. Standard errors are clustered at the school level. Longitudinal weights are used. *** p<0.01, ** p<0.05, * p<0.1.

Fig 3. Association between BMI Polygenic Scores and log(BMI) Along the Life-Cycle. HRS War Babies and Early Baby Boomers Cohorts

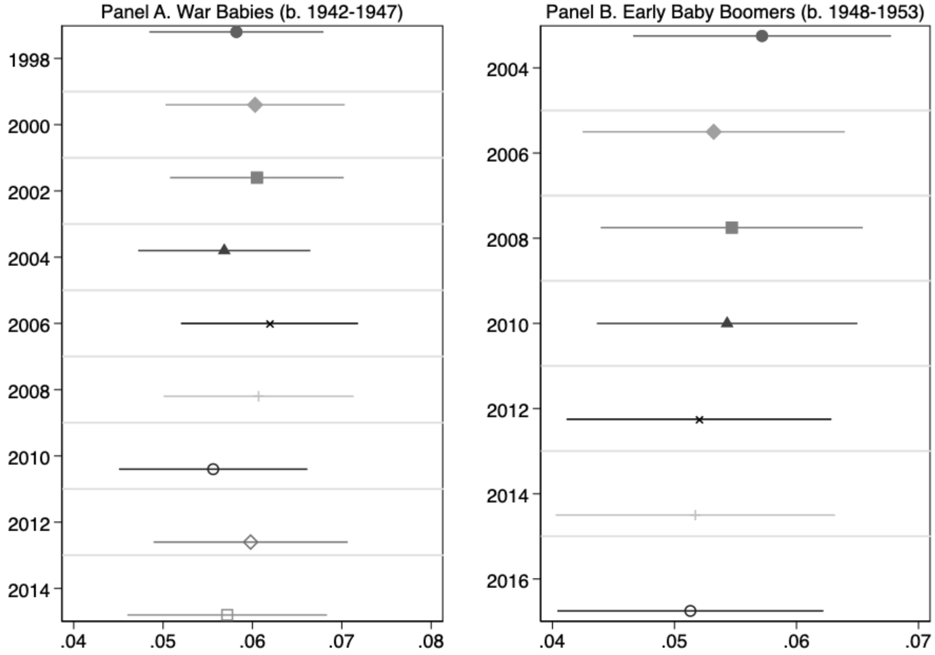

Note: This Figure summarizes the results of estimating equation 2 on two balanced samples of 1,201 HRS War Babies cohort members (Panel A) and 1,191 Early Baby Boomers cohort members (Panel B). The dependent variable is Log(BMI). OLS coefficient estimates of β_1 as well as their associated 95% confidence intervals are depicted. All regressions include a female dummy, age, age squared, and the first 10 principal components of the full matrix of genetic data. Standard errors are clustered at the household level.

Table 1. Association between BMI Polygenic Scores and Log(BMI) Along the Life-Cycle. HRS Original Cohort

	(1)<br>All	(2)<br>Low SES	(3)<br>High SES	(4)<br>Males	(5)<br>Females
1992: Age 55.9	0.041***	0.039***	0.043***	0.043***	0.039***
	(0.003)	(0.004)	(0.004)	(0.003)	(0.004)
1994: Age 57.8	0.040***	0.038***	0.040***	0.041***	0.038***
	(0.003)	(0.004)	(0.004)	(0.003)	(0.004)
1996: Age 59.8	0.042***	0.041***	0.043***	0.041***	0.043***
	(0.003)	(0.004)	(0.004)	(0.004)	(0.004)
1998:Age 61.6	0.041***	0.039***	0.043***	0.040***	0.041***
	(0.003)	(0.004)	(0.004)	(0.004)	(0.004)
2000: Age 63.6	0.041***	0.041***	0.041***	0.042***	0.041***
	(0.003)	(0.004)	(0.004)	(0.004)	(0.004)
2002: Age 65.7	0.041***	0.041***	0.041***	0.041***	0.041***
	(0.003)	(0.004)	(0.004)	(0.004)	(0.004)
2004: Age 67.8	0.043***	0.042***	0.043***	0.042***	0.043***
	(0.003)	(0.004)	(0.004)	(0.004)	(0.005)
2006: Age 69.7	0.042***	0.040***	0.044***	0.041***	0.043***
	(0.003)	(0.004)	(0.004)	(0.004)	(0.004)
2008: Age 71.7	0.042***	0.039***	0.044***	0.042***	0.041***
	(0.003)	(0.004)	(0.004)	(0.004)	(0.005)
Observations	3,181	1,567	1,614	1,423	1,758

Note: The dependent variable is Log(BMI). The Table displays OLS coefficient estimates of $BMIPGS$ (normalized to have mean 0 and standard deviation 1) in equation 2. The benchmark sample used for these analyses is described in Table 1 of the main text. All regressions include age, age squared, and the first 10 principal components of the full matrix of genetic data as controls. In Columns 1-3 a female dummy is also included as a regressor. The samples used in Columns 2 and 3 include individuals whose parental socioeconomic status is below and above the median, respectively. Standard errors (in parentheses) are clustered at the household level. *** p<0.01, ** p<0.05, * p<0.1.

Table 2. Association between BMI Polygenic Scores and Log(BMI) Along the Life-Cycle. Add Health Cohort

	(1) All	(2) Low SES	(3) High SES	(4) Males	(5) Females
1994/95: Age 15.4	0.042***	0.054***	0.026***	0.041***	0.044***
	(0.005)	(0.006)	(0.006)	(0.006)	(0.006)
1996: Age 16.3	0.046***	0.062***	0.023***	0.045***	0.046***
	(0.006)	(0.007)	(0.007)	(0.007)	(0.007)
2001/02: Age 21.7	0.049***	0.063***	0.030***	0.047***	0.052***
	(0.006)	(0.008)	(0.007)	(0.007)	(0.009)
2008/09: Age 28.3	0.057***	0.073***	0.033***	0.051***	0.063***
	(0.006)	(0.007)	(0.009)	(0.007)	(0.010)
2016/18: Age 37.3	0.058***	0.068***	0.039***	0.057***	0.056***
	(0.006)	(0.007)	(0.009)	(0.007)	(0.009)
Observations	2,730	1,361	1,369	1,204	1,526

Note: The dependent variable is Log(BMI). The Table displays OLS coefficient estimates of $BMIPGS$ (normalized to have mean 0 and standard deviation 1) in equation 2. The benchmark sample used for these analyses is described in Table 2 of the main text. All regressions include age, age squared, and the first 10 principal components of the full matrix of genetic data as controls. In Columns 1-3 a female dummy is also included as a regressor. The samples used in Columns 2 and 3 include individuals whose parental socioeconomic status is below and above the median, respectively. Standard errors (in parentheses) are clustered at the school level. Longitudinal weights are used. *** p<0.01, ** p<0.05, * p<0.1.

Table 3. Association between BMI Polygenic Scores and Log(BMI) Along the Life-Cycle and Pubertal Stage. Add Health Cohort

	(1) Benchmark	(2) With Controls for Pubertal Stage
1994/95: Age 15.4	0.042***	0.039***
	(0.005)	(0.004)
1996: Age 16.3	0.046***	0.038***
	(0.006)	(0.006)
2001/02: Age 21.7	0.049***	0.049***
	(0.006)	(0.006)
2008/09: Age 28.3	0.057***	0.057***
	(0.006)	(0.006)
2016/18: Age 37.3	0.058***	0.058***
	(0.006)	(0.006)
Observations	2,730	2,730

Note: The dependent variable is Log(BMI). The Table displays OLS coefficient estimates of $BMIPGS$ (normalized to have mean 0 and standard deviation 1) in equation 2. All specifications include the following covariates: a female dummy, age, age squared, and the first 10 principal components of the full matrix of genetic data. The specifications for Waves I (1994/95) and II (1996) in Column (2) also include gender and wave specific controls for pubertal stage. Standard errors (in parentheses) are clustered at the school level. Longitudinal weights are used. *** p<0.01, ** p<0.05, * p<0.1.

Table 4. Association between BMI Polygenic Scores and Log(BMI) Along the Life-Cycle and Pubertal Timing. Add Health Cohort

	(1)<br>Benchmark	(2)<br>Puberty Onset Timing Among Controls
1994/95: Age 15.4	0.042***	0.041***
	(0.005)	(0.005)
1996: Age 16.3	0.046***	0.044***
	(0.006)	(0.006)
2001/02: Age 21.7	0.049***	0.048***
	(0.006)	(0.006)
2008/09: Age 28.3	0.057***	0.055***
	(0.006)	(0.006)
2016/18: Age 37.3	0.058***	0.056***
	(0.006)	(0.006)
Observations	2,730	2,708

Note: The dependent variable is Log(BMI). The Table displays OLS coefficient estimates of BMIPGS (normalized to have mean 0 and standard deviation 1) in equation 2. All specifications include the following covariates: a female dummy, age, age squared, and the first 10 principal components of the full matrix of genetic data. An indicator for early vs. delayed puberty onset is added in Column 2. Standard errors (in parentheses) are clustered at the school level. Longitudinal weights are used. *** p<0.01, ** p<0.05, * p<0.1.

Table 5. Association between BMI Polygenic Scores and Log(BMI) Along the Life-Cycle and the Prevalence of Chronic Diseases. HRS Original Cohort

	(1) Benchmark	(2) With Controls for Chronic Diseases
1992: Age 55.9	0.041***	0.039***
	(0.003)	(0.003)
1994: Age 57.8	0.040***	0.038***
	(0.003)	(0.003)
1996: Age 59.8	0.042***	0.039***
	(0.003)	(0.003)
1998:Age 61.6	0.041***	0.038***
	(0.003)	(0.003)
2000: Age 63.6	0.041***	0.038***
	(0.003)	(0.003)
2002: Age 65.7	0.041***	0.038***
	(0.003)	(0.003)
2004: Age 67.8	0.043***	0.039***
	(0.003)	(0.003)
2006:Age 69.7	0.042***	0.037***
	(0.003)	(0.003)
2008: Age 71.7	0.042***	0.036***
	(0.003)	(0.003)
Observations	3,181	3,161

Note: The dependent variable is Log(BMI). The Table displays OLS coefficient estimates of BMIPGS (normalized to have mean 0 and standard deviation 1) in equation 2. All regressions include a female dummy, age, age squared, and the first 10 principal components of the full matrix of genetic data. The specification in Column (2) adds period specific indicators for the prevalence of the following diseases: cancer, lung disease, heart disease, diabetes, and arthritis. Standard errors (in parentheses) are clustered at the household. *** $p<0.01$, ** $p<0.05$, * $p<0.1$.

Table 6. Association between BMI Polygenic Scores and Obesity Along the Life-Cycle. HRS Original Cohort. Correction for Mortality Selection Using Inverse Probability Weights

	(1)<br>All	(2)<br>Low SES	(3)<br>High SES	(4)<br>Males	(5)<br>Females
1992: Age 55.9	0.041***	0.040***	0.043***	0.043***	0.040***
	(0.003)	(0.004)	(0.004)	(0.003)	(0.004)
1994: Age 57.8	0.040***	0.039***	0.040***	0.041***	0.039***
	(0.003)	(0.004)	(0.004)	(0.003)	(0.004)
1996: Age 59.8	0.043***	0.042***	0.043***	0.041***	0.044***
	(0.003)	(0.004)	(0.004)	(0.004)	(0.004)
1998:Age 61.6	0.041***	0.040***	0.042***	0.040***	0.042***
	(0.003)	(0.004)	(0.004)	(0.004)	(0.004)
2000: Age 63.6	0.042***	0.042***	0.041***	0.042***	0.042***
	(0.003)	(0.004)	(0.004)	(0.004)	(0.004)
2002: Age 65.7	0.041***	0.042***	0.041***	0.040***	0.042***
	(0.003)	(0.004)	(0.004)	(0.004)	(0.004)
2004: Age 67.8	0.043***	0.043***	0.043***	0.042***	0.044***
	(0.003)	(0.004)	(0.004)	(0.004)	(0.005)
2006:Age 69.7	0.042***	0.041***	0.044***	0.040***	0.044***
	(0.003)	(0.004)	(0.004)	(0.004)	(0.004)
2008: Age 71.7	0.042***	0.040***	0.044***	0.040***	0.043***
	(0.003)	(0.005)	(0.004)	(0.004)	(0.005)
Observations	3,181	1,567	1,614	1,423	1,758

Note: The dependent variable is Log(BMI). The Table displays OLS coefficient estimates of $BMIPGS$ (normalized to have mean 0 and standard deviation 1) in equation 2. The benchmark sample used for these analyses is described in Table 1 of the main text. All regressions include age, age squared, and the first 10 principal components of the full matrix of genetic data as controls. In Columns 1-3 a female dummy is also included as a regressor. The samples used in Columns 2 and 3 include individuals whose parental socioeconomic status is below and above the median, respectively. In these estimations we have corrected for selective mortality using inverse probability weights as described in Section Attrition. Standard errors (in parentheses) are clustered at the household level. *** p<0.01, ** p<0.05, * p<0.1.

Table 7. Association between BMI Polygenic Scores and Log(BMI) Along the Life-Cycle. BMI based on Objective Measurements vs. Self-Reports

	(1)<br>Objective	(2)<br>Self-Reported
	Panel A: HRS Original Cohort	
2006: Age 69.7	0.044***	0.042***
	(0.005)	(0.003)
2008: Age 71.7	0.044***	0.042***
	(0.005)	(0.003)
	Panel B: Add Health Cohort	
1996: Age 16.3	0.046***	0.046***
	(0.005)	(0.006)
2001/02: Age 21.7	0.053***	0.049***
	(0.006)	(0.006)
2008/09: Age 28.3	0.059***	0.057***
	(0.006)	(0.006)
2016/18: Age 37.3	0.069***	0.058***
	(0.009)	(0.006)

Note: The dependent variables are Log(BMI) based on objective measurements (Column 1) and self reports (Column 2), respectively. The Table displays OLS coefficient estimates of BMIPGS (normalized to have mean 0 and standard deviation 1) in equation 2. All regressions include a female dummy, age, age squared, and the first 10 principal components of the full matrix of genetic data. Standard errors (in parentheses) are clustered at the household (Panel A) and school (Panel B) level, respectively. Longitudinal weights are used in Panel A. *** $p<0.01$, ** $p<0.05$, * $p<0.1$.

Table 8. Association between BMI Polygenic Scores, Childhood Socioeconomic Status, and Log(BMI) Along the Life-Cycle. Add Health Cohort

	(1)<br>With SES Included as Control	(2)	(3)<br>Benchmark
	BMI PGS	SES	BMI PGS
1994/95: Age 15.4	0.041***	-0.017***	0.042***
	(0.005)	(0.006)	(0.005)
1996: Age 16.3	0.044***	-0.019**	0.046***
	(0.005)	(0.007)	(0.006)
2001/02: Age 21.7	0.048***	-0.020***	0.049***
	(0.006)	(0.007)	(0.006)
2008/09: Age 28.3	0.055***	-0.027***	0.057***
	(0.006)	(0.008)	(0.006)
2016/18: Age 37.3	0.055***	-0.034***	0.058***
	(0.006)	(0.007)	(0.006)
Observations	2,730		2,730

Note: The dependent variable is Log(BMI). The Table displays OLS coefficient estimates of BMIPGS and childhood SES (both normalized to have mean 0 and standard deviation 1). All specifications include the following covariates: a female dummy, age, age squared, and the first 10 principal components of the full matrix of genetic data. The specification used in Columns 1 and 2 adds childhood SES as an additional covariate. Standard errors (in parentheses) are clustered at the school level. Longitudinal weights are used. *** $p<0.01$, ** $p<0.05$, * $p<0.1$.

Table 9. Association between BMI Polygenic Scores, Childhood Socioeconomic Status, and Log(BMI) Along the Life-Cycle. HRS Original Cohort

	(1)	(2)	(3)
	With SES Included as Control		Benchmark
	BMI PGS	SES	BMI PGS
1992: Age 55.9	0.041***	-0.010*	0.041***
	(0.003)	(0.004)	(0.003)
1994: Age 57.8	0.039***	-0.010*	0.040***
	(0.003)	(0.004)	(0.003)
1996: Age 59.8	0.042***	-0.012**	0.042***
	(0.003)	(0.004)	(0.003)
1998:Age 61.6	0.041***	-0.010*	0.041***
	(0.003)	(0.005)	(0.003)
2000: Age 63.6	0.041***	-0.013**	0.041***
	(0.003)	(0.005)	(0.003)
2002: Age 65.7	0.041***	-0.012**	0.041***
	(0.003)	(0.005)	(0.003)
2004: Age 67.8	0.042***	-0.012**	0.043***
	(0.003)	(0.005)	(0.003)
2006:Age 69.7	0.042***	-0.010*	0.042***
	(0.003)	(0.005)	(0.003)
2008: Age 71.7	0.041***	-0.011*	0.042***
	(0.003)	(0.005)	(0.003)
Observations	3,181		3,181

Note: The dependent variable is Log(BMI). The Table displays OLS coefficient estimates of BMIPGS and childhood SES (both normalized to have mean 0 and standard deviation 1). All specifications include the following covariates: a female dummy, age, age squared, and the first 10 principal components of the full matrix of genetic data. The specification used in Columns 1 and 2 adds childhood SES as an additional covariate. Standard errors (in parentheses) are clustered at the household level. *** p<0.01, ** p<0.05, * p<0.1.

Childhood Socioeconomic Status Indices Construction

HRS

The HRS parental socioeconomic status index is based on several retrospective questions asked to survey participants that allow one to create the following variables:

1. Paternal education: years of schooling completed by respondents' fathers.
2. Family well off: an indicator variable identifying respondents who declare that their families were pretty well off or about average financially from birth to age 16.
3. Family never moved or asked for help for financial reasons: an indicator variable identifying respondents whose families ever had to move residence, or who never had to ask relatives for help due to financial reasons.
4. Father's employment: an indicator variable identifying respondents whose father never spent several months or more unemployed.

Next, we compute a summary index as in [1] that is equal to the unweighted average of the previous standardized variables:

$$SES^* = \frac{\sum_k SES_k^*}{K} \text{ , where } SES_k^* = \frac{SES_k - \mu_k}{\sigma_k}$$

, where SES_k is the kth component of the index, μ_k denotes its mean and σ_k its standard deviation. Since all the components are associated with higher socioeconomic status, higher values of the summary index SES^* are associated with higher socioeconomic status.

Add Health

To measure childhood SES in Add Health we construct an index based on parental education, parental occupation prestige, household income, and household receipt of public assistance following [2] and [3]. We have constructed an occupational prestige indicator using occupational prestige scores from the National Opinion Research Center (NORC) occupational classification. See `http://ibgwww.colorado.edu/~agross/NNSD/prestige%20scores.html`. The information we use was gathered at Wave I. Some of the variables used are based on questions included in the parental questionnaire (household income), while others rely on adolescents' responses (parental occupation and household receipt of public assistance). In some cases (parental education) we have complemented information based on questions addressed to parents with information based on adolescents' answers.

Finally, we conducted principal components analysis of parental education, parental occupational attainment, family income, and household receipt of public assistance to produce a factor score. The first principal component explained 49.2% of the variance. We used loadings on this component to compute a SES index, and then we standardized it to have mean 0 and standard deviation 1.

References

1. Kling JR, Liebman JB, Katz LF. Experimental analysis of neighborhood effects. Econometrica. 2007;75(1):83–119.

2. Belsky DW, Domingue BW, Wedow R, Arseneault L, Boardman JD, Caspi A, et al. Genetic analysis of social-class mobility in five longitudinal studies. Proceedings of the National Academy of Sciences. 2018;115(31):E7275–E7284.

3. Sanz-de Galdeano A, Terskaya A. Sibling Differences in Educational Polygenic Scores: How Do Parents React? IZA Discussion Paper No. 12375; 2019.
