# Supplementary figures and images for: Association of a genetic risk score with BMI along the life-cycle: Evidence from several US cohorts
Source: PLoS One. 2020 Sep 17;15(9):e0239067. doi: 10.1371/journal.pone.0239067 (PMC7497990; doi:10.1371/journal.pone.0239067)

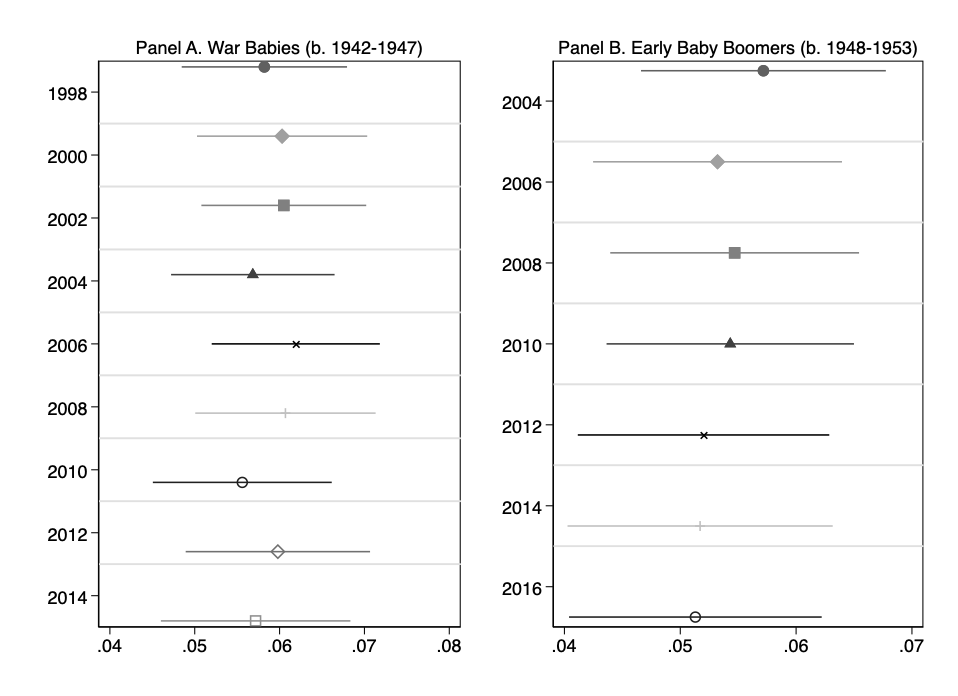

Supplement: S1 File — (ZIP) [file pone.0239067.s002.zip › HRS_ACohorts.png]

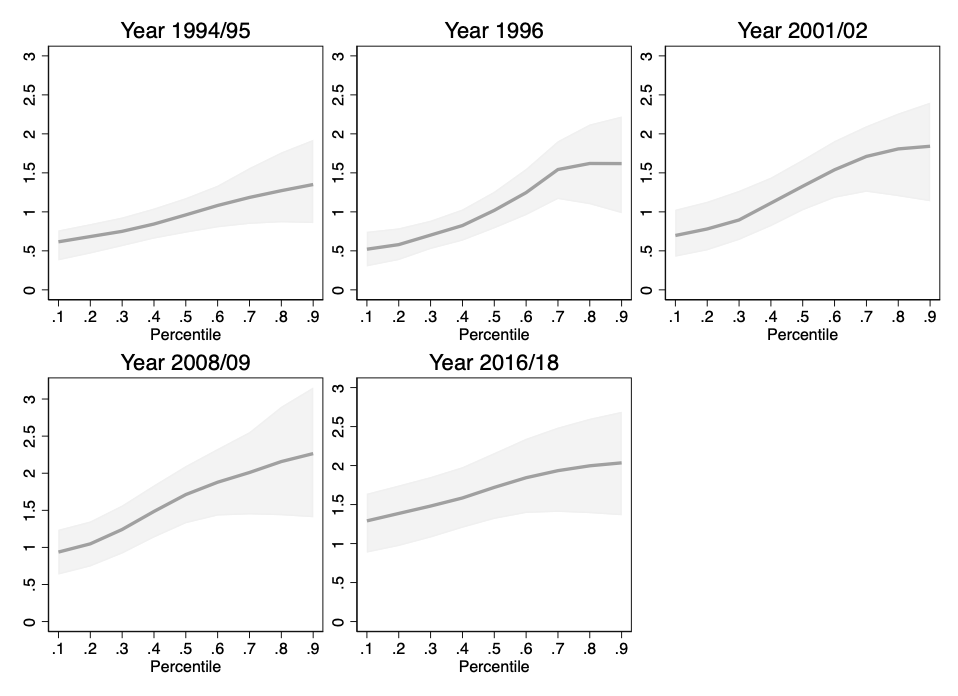

Supplement: S1 File — (ZIP) [file pone.0239067.s002.zip › rifregBMI_AH.png]

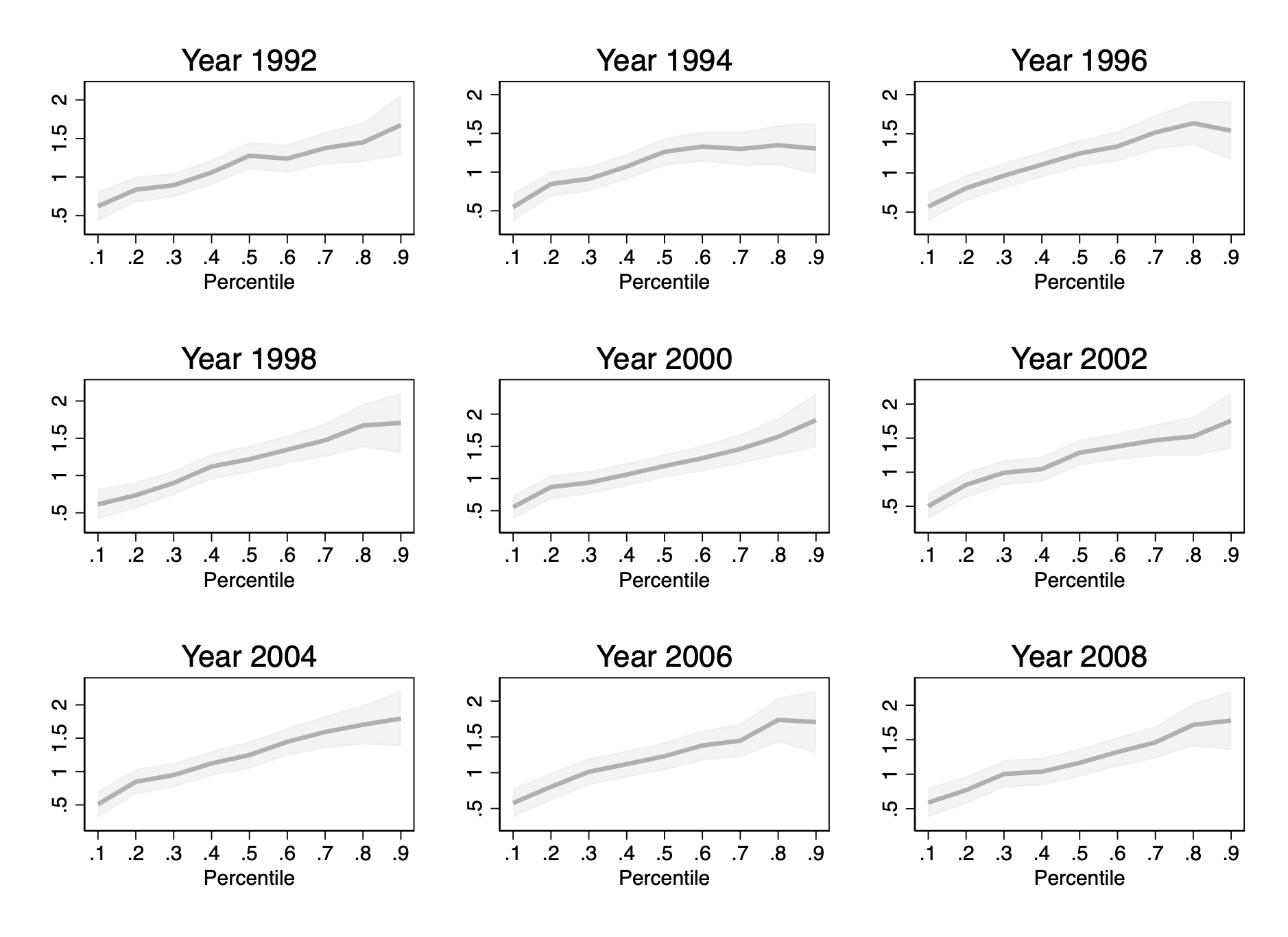

Supplement: S1 File — (ZIP) [file pone.0239067.s002.zip › rifregBMI_HRS.png]
