# Supplementary material for: Association of a genetic risk score with BMI along the life-cycle: Evidence from several US cohorts
Source: PLoS One. 2020 Sep 17;15(9):e0239067. doi: 10.1371/journal.pone.0239067 (PMC7497990; doi:10.1371/journal.pone.0239067)
Supplement: Attachment — Submitted filename: Response to Reviewers.pdf [file pone.0239067.s003.pdf]

Ref: PONE-D-20-03465

Association of a Genetic Risk Score with BMI along the Life-Cycle: Evidence from several US Cohorts

PLOS ONE

April 21st, 2020

Dear Referees,

Please find enclosed a revised version of the paper, Manuscript Ref PONE-D-20-03465, “Association of a Genetic Risk Score with BMI along the Life-Cycle: Evidence from several US Cohorts”. We thank you and the Editor for your comments, and for giving us the opportunity to revise the paper. We believe the paper has considerably improved as a result. Further details are in our point-to-point reply letters that follow.

Yours sincerely,
The authors

Reviewer #1: This article reports analysis of genetic association with BMI across the life course. The authors ask if genetic associations with BMI are stronger at some ages as compared to others. They use data from two large social surveys that include longitudinal assessments of BMI. The main finding is that genetic associations with BMI are somewhat stronger at older ages up to the 50s/60s, when associations stabilize.

These findings are consistent with previous reports in both the twin and molecular genetic literatures. The authors should note the Belsky et al. 2012 paper, which was the first to report polygenic score associations with BMI development across the first half of the life course. (the Hardy et al. paper cited analyzed only the FTO polymorphism)

Thank you for bringing the Belsky et al. (2012) study to our attention. We now refer to it in the Introduction. They use the Dunedin Multidisciplinary Health and Development Study, which followed individuals born in 1972-73 in Dunedin (New Zealand) from birth through 38 years. They find that higher BMI polygenic scores predict higher BMI growth during childhood (from ages 3 through 13 years), as well as during adulthood (from ages 13 through 38 years). Our US evidence based on the Add Health cohort is actually aligned with their results for ages 13 through 38 years, as we find that the association between BMI PGS and BMI increases as adolescents transition and settle into adulthood. Additionally, we show that the life-cycle pattern of BMI PGS-BMI association is stable among HRS Original cohort members as they transition from middle-age to old-age.

The main technical criticism I would make of this analysis is that the authors do not take seriously enough the limitation that much of their data come from self-reports of height and weight. They tell us they did some analysis to evaluate the risk this posed to the inferences they make but don't show the data. They should.

This is particularly important for interpreting genetic associations with BMI across the transition from adolescence to young adulthood.

The adolescent BMI data come from self-reports whereas the young adult data come from anthropometric assessments.

If there is less error in measurement in the anthropometry-derived BMIs, this will lead to larger effect-sizes in association analysis.

Thank you for raising these points. We acknowledge the previous version of our manuscript did not properly reflect the importance of the potential issues related to the fact that our benchmark analyses rely on self-reported data. The revised version of the paper has improved in this regard. We summarize our main amendments below:

First, we have clarified that <u>all</u> our benchmark analyses (not just those referred to adolescent BMI data) are based on self-reported data. We deliberately made this choice precisely because we did not want to have different BMI measurements (self-reported or objective) for different ages. Add Health objective measures are available

in Waves II-V (not in Wave I though, when individuals were 16.3 years old on average), while HRS objective measures are only available after 2006.

Second, we now show, as you request, estimation results based on objective BMI measures (whenever available), and compare them with our benchmark results based on subjective BMI measures. These results are reported in Table R1 below and in S1 Appendix Table 7 (Section Objective Measurements versus Self-Reports of Weight and428Height) of the revised version of the paper.

Panel A in Table R1 displays the estimated associations between BMI PGS and objective (Column 1) and self-reported (Column 2) log(BMI) for the HRS Original cohort for years 2006 and 2008 (our sample years with available objective BMI measures). The comparison of Columns 1 and 2 reveals that the estimated associations between BMI PGS and objective and self-reported log(BMI) barely differ. Therefore, our conclusion that the link between BMI PGS and log(BMI) is stable over as middle-age individuals transition to old-age remains when using objective BMI measures.

Panel B in Table R1 does the same comparative analysis for the Add Health cohort. The estimated coefficients of BMI PGS do not significantly differ (at the 5% level) across columns for all waves. Importantly, our finding that the association between BMI PGS and log(BMI) increases as adolescents transition into adulthood prevails when using objective BMI measures.

Table R1: Association between BMI Polygenic Scores and Log(BMI) Along the Life-Cycle. BMI based on Objective Measurements vs. Self-Reports.

	(1)<br>Objective	(2)<br>Self-Reported
Panel A: HRS Original Cohort		
2006: Age 69.7	0.044***	0.042***
	(0.005)	(0.003)
2008: Age 71.7	0.044***	0.042***
	(0.005)	(0.003)
Panel B: Add Health Cohort		
1996: Age 16.3	0.046***	0.046***
	(0.005)	(0.006)
2001/02: Age 21.7	0.053***	0.049***
	(0.006)	(0.006)
2008/09: Age 28.3	0.059***	0.057***
	(0.006)	(0.006)
2016/18: Age 37.3	0.069***	0.058***
	(0.009)	(0.006)

Note: The dependent variables are *log*(BMI) based on objective measurements (Column 1) and self- reports (Column 2), respectively. The Table displays OLS coefficient estimates of BMIPGS (normalized to have mean 0 and standard deviation 1) in equation 2. All regressions include a female dummy, age, age squared, and the first 10 principal components of the full matrix of genetic data. Standard errors (in parentheses) are clustered at the household (Panel A) and school (Panel B) level, respectively. Longitudinal weights are used in Panel A. *** $p<0.01$, ** $p<0.05$, * $p<0.1$.

A second critique is that the authors don't seem to think much about the biology of BMI change across the life course and how this may affect genetic associations. Two processes are of particular relevance to the analysis reported. First, puberty causes substantial changes in BMI. Pubertal timing varies across individuals. Variation in pubertal timing may therefore result in a kind of measurement error in the BMI phenotype being analyzed in adolescence, biasing genetic effect-sizes toward the null.

Second, with advancing age, a range of chronic diseases become more prevalent, leading to wasting (BMI loss). Add Health data on timing of menarche and HRS data on chronic disease morbidity may be helpful in exploring these processes.

Thank you for your suggestion. We have exploited the information available in both data sets to investigate these issues.

As you point out puberty and BMI are likely related (Ong et. al., 2012; Solorzano and McCartney, 2010, among others), and pubertal timing differs across individuals. Therefore, part of BMI variation during adolescence may be due to pubertal stage differences across teenage respondents. Hence, the variance of the error in equation (2) is likely larger for adolescents than for older individuals. Moreover, there is evidence that pubertal timing and BMI have a common genetic component and therefore part of the effect of genes on BMI might be explained by the effect of genes on pubertal timing (Elks et. al., 2010, Day et. al., 2017).

To address these points, we have replicated our baseline analyses including gender-specific information on the stage of development of adolescents that Add Health collected in Waves I and II, as by Wave III individuals were already between 18 and 26 years old (21.7 years old on average in our analytic sample).

In particular, we use the following questions that were asked to boys in Waves I and II: i) "How much hair is under your arms now? 1 I have no hair at all, 2 I have a little hair, 3 I have some hair, but not a lot; it has spread out since it first started, 4 I have a lot of hair that is thick, 5 I have a whole lot of hair that is very thick, as much hair as a grown man"; ii) "How thick is the hair on your face? 1 I have a few scattered hairs, but the growth is not thick, 2 The hair is somewhat thick, but you can still see a lot of skin under it, 3 The hair is thick; you can't see much skin under it, 4 The hair is very thick, like a grown man's facial hair"; iii) "Is your voice lower now than it was when you were in grade school? 1 No, it is about the same as when you were in grade school, 2 Yes, it is a little lower than when you were in grade school, 3 Yes, it is somewhat lower than when you were in grade school, 4 Yes, it is a lot lower than when you were in grade school, 5 Yes, it is a whole lot lower than when you were in grade school; it is as low as an adult man's voice"; and iv) "How advanced is your physical development compared to other boys your age? 1 I look younger than most, 2 I look younger than some, 3 I look about average, 4 I look older than some, 5 I look older than most".

As for girls, we use the following questions that were asked in Waves I and II: i) "As a girl grows up her breasts develop and get bigger. Which sentence best describes you? 1 My breasts are about the same size as when I was in grade school, 2 My

breasts are a little bigger than when I was in grade school, 3 My breasts are somewhat bigger than when I was in grade school, 4 My breasts are a lot bigger than when I was in grade school, 5 My breasts are a whole lot bigger than when I was in grade school, they are as developed as a grown woman's breasts"; ii) "As a girl grows up her body becomes more curved. Which sentence best describes you? 1 My body is about as curvy as when I was in grade school, 2 My body is a little more curvy than when I was in grade school, 3 My body is somewhat more curvy than when I was in grade school, 4 My body is a lot more curvy than when I was in grade school, 5 My body is a whole lot more curvy than when I was in grade school"; iii) "Have you ever had a menstrual period (menstruated)? 0 No, 1 Yes"; and iv) "How advanced is your physical development compared to other girls your age? 1 I look younger than most, 2 I look younger than some, 3 I look about average, 4 I look older than some, 5 I look older than most".

We construct binary indicators for all the possible answers to these questions and we add them as controls to our estimations of equation (2) for Waves I and II. The results of this analysis, reported in Table R2 (and in Table 3 in S1 Appendix, discussed in Section Pubertal Stage and the Association of BMI PGS with BMI of the revised manuscript), indicate that the effect of BMI PGS on log(BMI) is lower after the inclusion of puberty stage controls. This is consistent with the fact that pubertal timing and BMI have a common genetic component. As a consequence, the estimated association between BMI PGS and log(BMI) increases more markedly as individuals transition from adolescence into adulthood when we control for pubertal stage indicators than when we do not (see Figure 3 and S1 Appendix Table 2). While it is reassuring that our conclusion is robust to the addition of pubertal stage indicators, our preferred specification excludes this set of controls in order to avoid reverse causality bias, as there is evidence that childhood obesity increases the risk of premature puberty for girls and boys (Solorzano and McCartney, 2010).

Moreover, we have re-estimated our benchmark model including pubertal timing as an additional regressor in Table R3 (Table 4 in S1 Appendix, Section Pubertal Stage and the Association of BMI PGS with BMI of the revised manuscript). Females' puberty onset is classified as early vs. delayed if age of menarche was lower than 13 (which is the median in our sample) vs. 13+. Establishing males' puberty onset is more complex. We do so following the recommendations from Mendle et al. (2019). In particular, we regress a pubertal status index on age, and we then save the residuals. The pubertal status index has been constructed using principal component analysis on the variables related to pubertal stage for boys previously described and measured in Wave I, as they display more variation in Wave I than in Wave II. Males' puberty onset is subsequently classified as early vs. delayed if the regression's residuals are below vs. above the median. As the comparison between Columns 1 and 2 of Table R3 reveals, the inclusion of pubertal timing as a control barely alters the estimated coefficients of BMI PGS.

Table R2: Association between BMI Polygenic Scores and Log(BMI) Along the Life-Cycle and Pubertal Stage. Add Health Cohort

	(1)<br>Benchmark	(2)<br>With Controls for Pubertal Stage
1994/95: Age 15.4	0.042***	0.039***
	(0.005)	(0.004)
1996: Age 16.3	0.046***	0.038***
	(0.006)	(0.006)
2001/02: Age 21.7	0.049***	0.049***
	(0.006)	(0.006)
2008/09: Age 28.3	0.057***	0.057***
	(0.006)	(0.006)
2016/18: Age 37.3	0.058***	0.058***
	(0.006)	(0.006)
Observations	2,730	2,730

Note: The dependent variable is *log*(BMI). The Table displays OLS coefficient estimates of BMIPGS (normalized to have mean 0 and standard deviation 1) in equation 2. All specifications include the following covariates: a female dummy, age, age squared, and the first 10 principal components of the full matrix of genetic data. The specifications for Waves I (1994/95) and II (1996) in Column (2) also include gender and wave specific controls for pubertal stage. Standard errors (in parentheses) are clustered at the school level. Longitudinal weights are used. *** p<0.01, ** p<0.05, * p<0.1.

Table R3: Association between BMI Polygenic Scores and Log(BMI) Along the Life-Cycle and Pubertal Timing. Add Health Cohort

	(1)<br>Benchmark	(2)<br>Puberty Onset Timing Among Controls
1994/95: Age 15.4	0.042***	0.041***
	(0.005)	(0.005)
1996: Age 16.3	0.046***	0.044***
	(0.006)	(0.006)
2001/02: Age 21.7	0.049***	0.048***
	(0.006)	(0.006)
2008/09: Age 28.3	0.057***	0.055***
	(0.006)	(0.006)
2016/18: Age 37.3	0.058***	0.056***
	(0.006)	(0.006)
Observations	2,730	2,708

Note: The dependent variable is *log*(BMI). The Table displays OLS coefficient estimates of BMIPGS (normalized to have mean 0 and standard deviation 1) in equation 2. All specifications include the following covariates: a female dummy, age, age squared, and the first 10 principal components of the full matrix of genetic data. An indicator for early vs. delayed puberty onset is added in Column 2. Standard errors (in parentheses) are clustered at the school level. Longitudinal weights are used. *** p<0.01, ** p<0.05, * p<0.1.

In summary, this evidence indicates that the increasing pattern of association between BMI PGS and log(BMI) we find for Add Health adolescents as they transition into adulthood is robust to the inclusion of controls for pubertal stage and the timing of puberty onset.

Regarding our HRS analyses, you point out that chronic diseases are more prevalent among the elderly, and they may in turn lead to wasting (BMI loss).

We have investigated whether our results are affected by the prevalence of the following conditions: heart disease, cancer, diabetes, lung disease, and arthritis. First, we have studied how the prevalence of this conditions correlates with both BMI and with BMI PGS in our analytic sample. The prevalence of heart disease, diabetes, and arthritis is positive and significantly correlated with BMI, while the prevalence of cancer, lung disease, and BMI are not significantly correlated. This pattern is the same for all sample years, that is, since individuals are on average 55.9 years old (in 1992) until they reach 71.7 years of age on average (in 2008). Hence, we find no evidence of BMI reductions being linked to higher prevalence of chronic diseases in our sample. The correlation between BMI PGS and chronic diseases is positive and significant .for heart disease, diabetes, and arthritis, while it is generally insignificant for cancer and lung disease.

Next, we replicate our baseline analyses including the prevalence of these five chronic conditions as additional controls in all our sample years. The results of this analysis, reported in Table R4 (Table 5 in S1 Appendix discussed in Section Morbidity and the Association of BMI PGS with BMI of the revised manuscript) reveal that the inclusion of this set of controls slightly attenuates the estimated association between BMI PGS and log(BMI). This is consistent with our previous finding that BMI PGS are positively and significantly correlated with several chronic diseases. Importantly, the life-cycle association between BMI PGS and log(BMI) remains stable as individuals transition from middle-age to old-age once these additional controls are included in our benchmark model (2). However, we do not include them in our preferred specification because their relationship with BMI is likely bidirectional.

Table R4: Association between BMI Polygenic Scores and Log(BMI) Along the Life-

Cycle and the Prevalence of Chronic Diseases. HRS Original Cohort		
	(1)	(2)
	Benchmark	With Controls for Chronic Diseases
1992: Age 55.9	0.041***	0.039***
	(0.003)	(0.003)
1994: Age 57.8	0.040***	0.038***
	(0.003)	(0.003)
1996: Age 59.8	0.042***	0.039***
	(0.003)	(0.003)
1998: Age 61.6	0.041***	0.038***
	(0.003)	(0.003)
2000: Age 63.6	0.041***	0.038***
	(0.003)	(0.003)
2002: Age 65.7	0.041***	0.038***
	(0.003)	(0.003)
2004: Age 67.8	0.043***	0.039***
	(0.003)	(0.003)
2006: Age 69.7	0.042***	0.037***
	(0.003)	(0.003)
2008: Age 71.7	0.042***	0.036***
	(0.003)	(0.003)
Observations	3181	3161

Note: The dependent variable is *log*(BMI). The Table displays OLS coefficient estimates of BMIPGS (normalized to have mean 0 and standard deviation 1) in equation 2. All regressions include a female dummy, age, age squared, and the first 10 principal components of the full matrix of genetic data. The specification in Column (2) adds period specific indicators for the prevalence of the following diseases: cancer, lung disease, heart disease, diabetes, and arthritis. Standard errors (in parentheses) are clustered at the household. *** p<0.01, ** p<0.05, * p<0.1.

Basically, my concern is that the authors are not identifying substantive differences in how genetics affect BMI, but instead are observing variation in the magnitudes of non-genetic causes (or genetic causes not measured in the PGS) across life course stages.

One idea to explore in evaluating this issue is to use some non-genetic measure of risk for obesity. For example, both Add Health and HRS measure parental education, which is associated with BMI across the life course. Do parental education associations with BMI show the same patterns of change with age as genetic associations?
If so, is this analysis telling us something about genetics or simply about the sources of systematic variation in BMI? If not, this is a strong piece of evidence that the patterning observed is specifically about the genetics being studied.

Thank you for raising this point. In order to investigate whether the life-cycle patterns of BMI PGS-log(BMI) associations we uncover systematically vary by childhood socioeconomic status (SES), we separately estimate our benchmark model (equation 2) for individuals from low and high SES backgrounds. As discussed in Section 3.2, we find that life-time patterns of association between BMI PGS and log(BMI) are similar for the low and high SES groups for the Add Health cohort members (whose

life-cycle pattern of genetic influence is increasing), and for the HRS Original cohort members (whose life-cycle pattern is stable).

Importantly, our SES index relies on very rich information on several parental background indicators including but not restricted to parental education (see Appendix XX for a detailed description of the construction of the childhood SES indices for the Add Health and the HRS Original cohorts).

In light of your comment, we have replicated our benchmark analyses including a childhood SES summary index among the set of control variables. This allows us to explore further whether the observed life-cycle associations between BMI PGS and log(BMI) reflect similar patterns of association between SES and log(BMI) as individuals grow older. The results of these analyses are shown in Tables R5 and R6 below (Tables 8 and 9 in S1 Appendix discussed in Section Socioeconomic Status and the Association of BMI PGS with BMI in the revised manuscript).

The association between SES and log(BMI) for Add Health cohort members is negative, significant, and it increases (in absolute terms) as they transition from adolescence into adulthood (Table R5, Column 2). However, the inclusion of SES among the control set barely changes the estimated coefficients of BMI PGS (Table R5, comparison of Columns 1 and 3). This indicates that SES effects across the life course cannot explain the observed increasing association between BMI PGS and log(BMI) between adolescence and early adulthood, which remains basically unaltered when SES is held constant.

The association between SES and log(BMI) for the HRS Original cohort members is negative and significant, and it does not significantly change as individuals get older (Table R6, Column 2). The inclusion of the childhood SES index among the set of control variables hardly modifies the estimated coefficients of BMI PGS (Table R6, comparison of Columns 1 and 3).

In summary, this evidence indicates that SES cannot account for the life-cycle patterns of association between BMI PGS and log(BMI) we have uncovered so far, neither for Add Health nor for HRS Original cohort members.

Table R5: Association between BMI Polygenic Scores, Childhood Socioeconomic

Status, and Log(BMI) Along the Life-Cycle. Add Health Cohort

	(1)	(2)	(3)
	With SES Included as Control		Benchmark
	BMI PGS (Normalized)	SES (Normalized)	BMI PGS (Normalized)
1994/95: Age 15.4	0.041***	-0.017***	0.042***
	(0.005)	(0.006)	(0.005)
1996: Age 16.3	0.044***	-0.019**	0.046***
	(0.005)	(0.007)	(0.006)
2001/02: Age 21.7	0.048***	-0.020***	0.049***
	(0.006)	(0.007)	(0.006)
2008/09: Age 28.3	0.055***	-0.027***	0.057***
	(0.006)	(0.008)	(0.006)
2016/18: Age 37.3	0.055***	-0.034***	0.058***
	(0.006)	(0.007)	(0.006)
Observations	2,730		2,730

Note: The dependent variable is *log*(BMI). The Table displays OLS coefficient estimates of BMIPGS and childhood SES (both normalized to have mean 0 and standard deviation 1). All specifications include the following covariates: a female dummy, age, age squared, and the first 10 principal components of the full matrix of genetic data. The specification used in Columns 1 and 2 adds childhood SES as an additional covariate. Standard errors (in parentheses) are clustered at the school level. Longitudinal weights are used. *** p<0.01, ** p<0.05, * p<0.1.

Table R6: Association between BMI Polygenic Scores, Childhood Socioeconomic Status, and Log(BMI) Along the Life-Cycle. HRS Original Cohort

	(1)	(2)	(3)
	With SES Included as Control		Benchmark
	BMI PGS (Normalized)	SES (Normalized)	BMI PGS (Normalized)
1992: Age 55.9	0.041***	-0.010*	0.041***
	(0.003)	(0.004)	(0.003)
1994: Age 57.8	0.039***	-0.010*	0.040***
	(0.003)	(0.004)	(0.003)
1996: Age 59.8	0.042***	-0.012**	0.042***
	(0.003)	(0.004)	(0.003)
1998: Age 61.6	0.041***	-0.010*	0.041***
	(0.003)	(0.005)	(0.003)
2000: Age 63.6	0.041***	-0.013**	0.041***
	(0.003)	(0.005)	(0.003)
2002: Age 65.7	0.041***	-0.012**	0.041***
	(0.003)	(0.005)	(0.003)
2004: Age 67.8	0.042***	-0.012**	0.043***
	(0.003)	(0.005)	(0.003)
2006: Age 69.7	0.042***	-0.010*	0.042***
	(0.003)	(0.005)	(0.003)
2008: Age 71.7	0.041***	-0.011*	0.042***

	(0.003)	(0.005)	(0.003)
Observations	3181		3181

Note: The dependent variable is *log*(BMI). The Table displays OLS coefficient estimates of BMIPGS and childhood SES (both normalized to have mean 0 and standard deviation 1). All specifications include the following covariates: a female dummy, age, age squared, and the first 10 principal components of the full matrix of genetic data. The specification used in Columns 1 and 2 adds childhood SES as an additional covariate. Standard errors (in parentheses) are clustered at the household level. *** p<0.01, ** p<0.05, * p<0.1.

MINOR

To my mind, there is a conceptual problem with the article. The authors approach their question within a GxE framework. But the changing association of genetics with BMI as people age is not a GxE. BMI growth is a developmental process, with BMI at later ages strongly influenced by BMI earlier on. Given the authors have repeated measures data on individuals, the question they should be asking is how do BMI genetics influence BMI change across the life course. But this is a matter of taste and differing views of how this problem should be approached ought not to interfere with publication in this journal.

Thank you for raising this point. We now avoid placing our contribution within the literature. We still refer to this literature because we present results for two different cohorts, and some authors have previously interpreted cohort differences as suggestive evidence that environmental factors affect genotype-phenotype associations. However, this is not our main focus, and ours is not a GxE paper.

Something else: The polygenic score analyzed by the authors comes from a GWAS that included mainly midlife individuals. For this reason, we might expect the strongest genetic associations in that age range. This might be discussed somewhat more in the introduction and discussion sections of the article.

Thank you for this point. We discuss this in the Conclusion, where we write:

Since the strength of genotype-phenotype associations may vary by age, GWAS results may not replicate in samples where the age distribution differs from that of the GWAS sample (Lasky-Su et al., 2008). As you point out, the BMI PGS we use rely on the GWAS conducted by Locke et al. (2015), which is in turn mostly based on a sample of midlife individuals. Hence, their predictive power may be lower for younger individuals. While the strongest BMI PGS-BMI association we uncover is for young adults (Waves 4 and 5 of Add Health), this warrants further investigation. As argued by Lasky-Su et al. (2008), using large longitudinal samples to discover age-varying genetic effects would be ideal because cross-sectional studies may fail to detect age-varying associations as they cannot disentangle age/time from cohort effects. A similar argument may apply to the predictive power of BMI PGS for individuals with different sociodemographic characteristics like childhood socioeconomic status (as our Add Health results by socioeconomic status suggest).

Reviewer #2: This is an excellent study, well performed and well written, and I recommend its publication.

Statistical methods are explained in detail and results are clear.

There is a typo in the abstract (a duplicated "the").

Congratulations.

Thank you very much for reading our work, we are very glad you liked it. We have corrected the typo you found in our abstract.

References *(not included in the paper initially submitted and added in this revision):*

Solorzano, C. M. B., & McCartney, C. R. (2010). Obesity and the pubertal transition in girls and boys. Reproduction (Cambridge, England), 140(3), 399.

Mendle, J., Beltz, A. M., Carter, R., & Dorn, L. D. (2019). Understanding puberty and its measurement: ideas for research in a new generation. Journal of Research on Adolescence, 29(1), 82-95.

Ong, K. K., Bann, D., Wills, A. K., Ward, K., Adams, J. E., Hardy, R., ... & National Survey of Health and Development Scientific and Data Collection Team. (2012). Timing of voice breaking in males associated with growth and weight gain across the life course. The Journal of Clinical Endocrinology & Metabolism, 97(8), 2844-2852.

Day, F. R., Thompson, D. J., Helgason, H., Chasman, D. I., Finucane, H., Sulem, P., ... & Altmaier, E. (2017). Genomic analyses identify hundreds of variants associated with age at menarche and support a role for puberty timing in cancer risk. Nature genetics, 49(6), 834-841.

Elks, C. E., Perry, J. R., Sulem, P., Chasman, D. I., Franceschini, N., He, C., ... & Gudbjartsson, D. F. (2010). Thirty new loci for age at menarche identified by a meta-analysis of genome-wide association studies. Nature genetics, 42(12), 1077.
