# Supplementary material for: Association of a genetic risk score with BMI along the life-cycle: Evidence from several US cohorts
Source: PLoS One. 2020 Sep 17;15(9):e0239067. doi: 10.1371/journal.pone.0239067 (PMC7497990; doi:10.1371/journal.pone.0239067)
Supplement: Attachment — Submitted filename: Response to Reviewers.docx [file pone.0239067.s004.docx]

**Ref: PONE-D-20-03465**

## Association of a Genetic Risk Score with BMI along the Life-Cycle: Evidence from several US Cohorts

## PLOS ONE

## July 26th, 2020

## Dear Referees,

## Please find enclosed a revised version of the paper, Manuscript Ref PONE-D-20-03465, “Association of a Genetic Risk Score with BMI along the Life-Cycle: Evidence from several US Cohorts”. We thank you and the Editor for your comments, and for giving us the opportunity to revise the paper. We believe the paper has considerably improved as a result. Further details are in our point-to-point reply letters that follow.

Yours sincerely,

The authors

**Thank you for submitting your manuscript to PLOS ONE. After careful consideration, we feel that it has merit but does not fully meet PLOS ONE’s publication criteria as it currently stands. Therefore, we invite you to submit a revised version of the manuscript that addresses the points raised during the review process.**

**More specifically, you must add a 'strengths and limitations' section in the discussion section of your manuscript.**

*We have added a section “strengths and limitations” in page 14, which includes the following points:*

1. *In this paper, we use two longitudinal surveys to provide new evidence on gene-age interaction effects on BMI for several cohorts. In particular, we study teenagers from the Add Health cohort (born 1974-1983) as they transition into adulthood as well as individuals aged 55+ who were born in earlier HRS cohorts (1931-53) as they move into old-age. The use of panel data is crucial in this context because it allows one to disentangle age/time associations from cohort effects. In contrast, as argued by (Lasky-Su et. al., 2008), cross-sectional studies may fail to detect age-varying associations as they cannot disentangle age/time from cohort effects.*

*Our analyses are based on different cohorts observed at different stages of the life cycle. Hence, our contrasting findings for Add Health and the HRS may reflect differing patterns of genetic influence along the life cycle, but they could also stem from systematic differences across cohorts in their life-cycle patterns of genetic influence.*

2. *Note also that in this paper we estimate a reduced-form model without digging into the mechanisms behind gene-age interactions because of data limitations. Our results therefore could be explained by changes in the biology of BMI across the life course as well as by environmental changes that may reinforce or mitigate the effect of genes on BMI (Sanz‐de‐Galdeano and Terskaya, 2019; Liu et. al. 2015; Barcellos et. al, 2018). Understanding the mechanisms behind the patterns we uncover is worth further investigation.*
3. *Another limitation of our analyses is that the genome-wide association study employed to compute the BMIPGS used mostly relies on European-descent individuals (Locke at. al., 2015). Therefore, our results cannot be generalized to individuals from different ancestries. The availability of GWAS for other ancestries would allow to overcome this limitation.*
4. *Finally, another potential limitation stems from the fact that the strength of genotype-phenotype associations may vary by age. Hence, GWAS results may not replicate in samples where the age distribution differs from that of the GWAS sample (Lasky-Su et. al., 2008). The BMIPGS we use rely on the GWAS conducted by Locke at. al. (2015). which is in turn mostly based on a sample of midlife individuals. Hence, their predictive power may be lower for younger individuals. A similar argument may apply to other demographic characteristics like childhood socioeconomic status (as our Add Health results by socioeconomic status suggest). While the strongest BMIPGS-BMI association we uncover is for young adults (Waves 4 and 5 of Add Health), this warrants further investigation.*

*Barcellos SH, Carvalho LS, Turley P. Education can reduce health differences related to genetic risk of obesity. Proceedings of the National Academy of Sciences. 2018;115(42):E9765-E9772.*

*Lasky-Su J, Lyon HN, Emilsson V, Heid IM, Molony C, Raby BA, et al. On the replication of genetic associations: timing can be everything! The American Journal of Human Genetics. 2008;82(4):849-858.*

*Liu H, Guo G. Lifetime socioeconomic status, historical context, and genetic inheritance in shaping body mass in middle and late adulthood. American sociological review. 2015;80(4):705-737.*

*Locke AE, Kahali B, Berndt SI, Justice AE, Pers TH, Day FR, et al. Genetic studies of body mass index yield new insights for obesity biology. Nature. 2015;518(7538):197.*

*Sanz-de Galdeano A, Terskaya A. Sibling Differences in Educational Polygenic Scores: How Do Parents React? IZA Discussion Paper No. 12375; 2019*
